# The transversely split gracilis twin free flaps

**DOI:** 10.4103/0970-0358.73435

**Published:** 2010

**Authors:** Divya N. Upadhyaya, Vaibhav Khanna, Surajit Bhattacharya, Sandeep Garg, Romesh Kohli

**Affiliations:** 1 Department of Plastic, Craniofacial and Microsurgery, Vivekananda Polyclinic and Institute of Medical Sciences, Vivekananda Puri, Mahanagar, India; 2 Department of Plastic, Reconstructive and Aesthetic Surgery, Sahara Hospital, Lucknow, India; 3 Department of Orthopedic Surgery and Joint Replacement, Vivekananda Polyclinic and Institute of Medical Sciences, Vivekananda Puri, Mahanagar, India; 4 Department of Orthopedic Surgery and Joint Replacement, Sahara Hospital, Lucknow, India

**Keywords:** Split gracilis muscle flap, gracilis flap, double muscle flap, one muscle two flaps

## Abstract

The gracilis muscle is a Class II muscle that is often used in free tissue transfer. The muscle has multiple secondary pedicles, of which the first one is the most consistent in terms of position and calibre. Each pedicle can support a segment of the muscle thus yielding multiple small flaps from a single, long muscle. Although it has often been split longitudinally along the fascicles of its nerve for functional transfer, it has rarely been split transversely to yield multiple muscle flaps that can be used to cover multiple wounds in one patient without subjecting him/her to the morbidity of multiple donor areas.

## INTRODUCTION

Trauma to the extremities often results in complex soft tissue defects requiring microsurgical reconstruction. The gracilis muscle is an infrequent choice for large soft tissue defects due to its diminutive size, but it can cover significantly large areas when its epimysium is cut and the muscle is “spread out.” The muscle has traditionally been used for functional transfers, small soft tissue defects like in the breast or the orbit, and coverage of exposed or osteomyelitic bone. It can also be split longitudinally according to the fascicular distribution of its nerve to suit the peculiar needs of a particular functional defect. It has an added advantage in that it is a Class II muscle with many secondary pedicles. These pedicles can each support a segment of the muscle, and one muscle may therefore yield multiple small flaps when split transversely. These split muscle flaps are of reliable vascularity with the added advantages of a single donor area.

## PATIENTS AND METHODS

**Figure 1:**
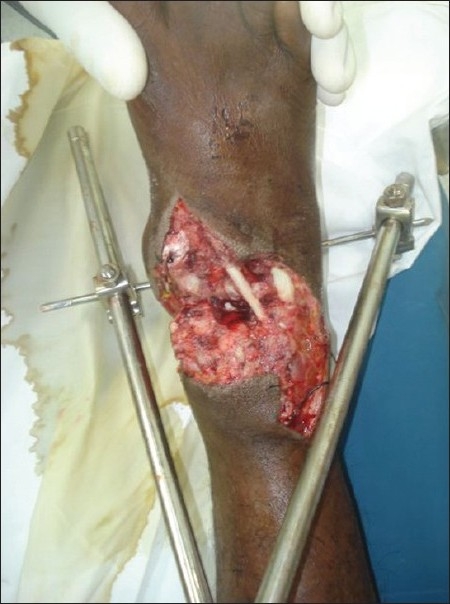
Right leg showing wound over the ankle

**Figure 2:**
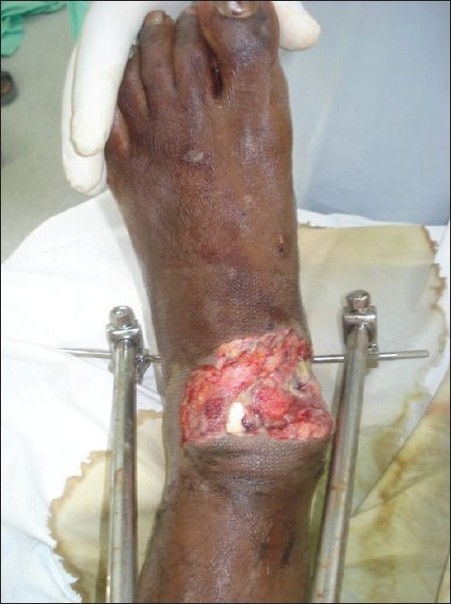
Left leg showing identical wound over the ankle

A young man aged 23 years was referred to the plastic surgery department from the orthopaedics department for the treatment of soft tissue defects over both ankles, having sustained trauma to both lower limbs in a motorcycle accident. After external fixator stabilisation of both lower limbs by the orthopaedic colleagues, the patient was referred to the plastic surgery department. On examination, the patient was found to have almost identical wounds over the dorsal aspect of both ankle joints, extending to almost half the circumference of the joint on the right side and from the dorsal midline to the medial malleolar area on the left side. Both the wounds showed exposed extensor tendons and distal tibia in the defect [Figures 1 and 2].

**Figure 3:**
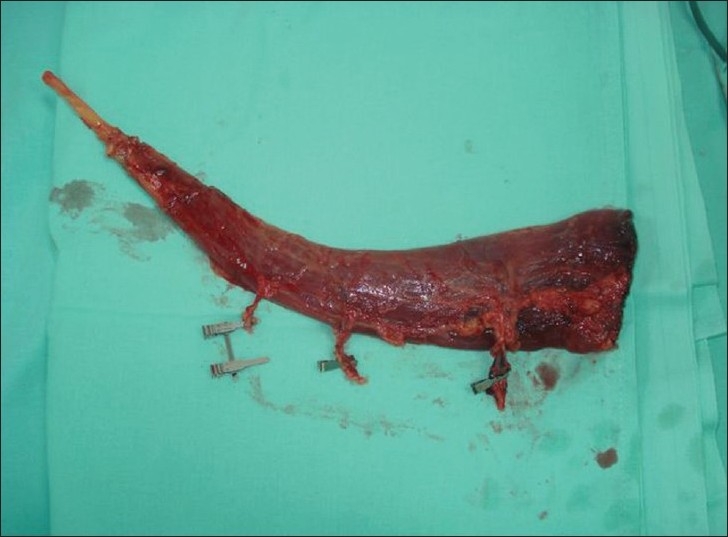
Harvested gracilis muscle

**Figure 4:**
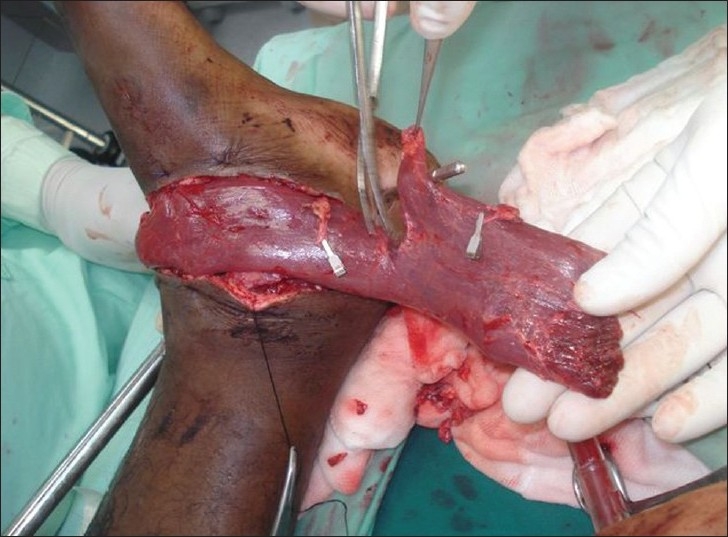
Gracilis muscle being split transversely

**Figure 5:**
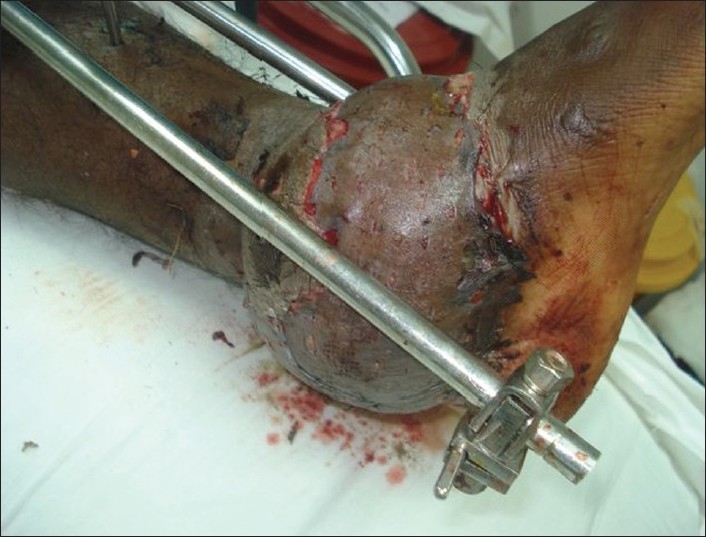
Early post operative wound left leg with good coverage and good take of the graft over the split gracilis muscle

**Figure 6:**
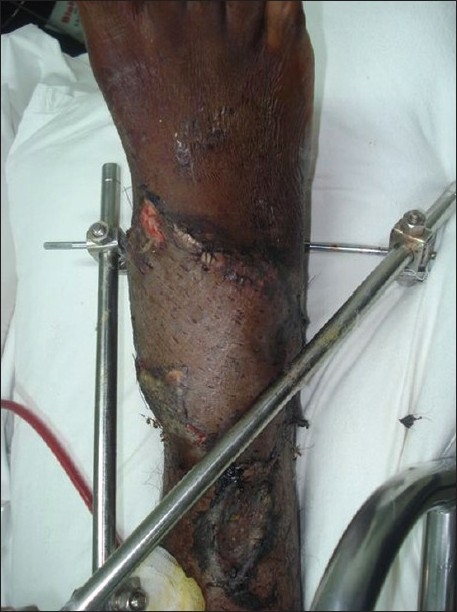
Early post operative result of the right leg shows stable wound coverage and good take of the skin graft

The patient was undertaken for surgery under epidural anaesthesia and tourniquet control for free flap cover of the wounds. The right gracilis muscle was harvested through a longitudinal incision in the medial thigh after flexing the knee joint and externally rotating the hip joint. The muscle was mobilised completely from its tendinous insertion to its origin from the pubic ramus. It was found to have one dominant and two minor pedicles [Figure 3]. The nerve supply to the muscle was ligated, cut and the proximal end was cauterised. The donor area was lavaged and closed over a suction drain. The harvested muscle was split transversely into two segments, each with a vascular pedicle of its own [Figure 4]. The twin flaps thus obtained were used to cover the identical defects of both legs. The artery of the muscle flap was anastomosed to the anterior tibial artery in an end-to-side fashion and the veins to the vena comitantes of the anterior tibial artery on their respective sides. The muscle flap was covered with a split-skin graft. The whole procedure lasted 4 h. The post operative course was uneventful [Figures 5 and 6] and the suction drain was removed after 48 hours. The patient was discharged on post op day 10 after removal of all the sutures.

## RESULTS

The patient was followed-up in the outpatient department at an interval of 3 weeks. On examination, the authors found a well-settled flap with a good take of the skin graft. There was no wound discharge and no sinuses. The flap had been successful in providing stable and well-vascularised coverage, resulting in quiet wound healing. The donor area scar was soft, supple, painless and well settled, with no tendency to hypertrophy. There was no functional deficit from harvesting of the gracilis muscle.

## DISCUSSION

Trauma to the extremities often results in complex injuries requiring free flap reconstruction. Various muscles have been used in extremity reconstruction since 1970. The gracilis muscle is usually not considered the first choice for free flap reconstruction in these defects.[1] and has historically been used for small soft tissue defects, reconstruction of head and neck deformities,[2] autologous breast reconstruction[3] and functional reconstruction of the face[4] and extremities.[5,6]

The gracilis muscle is a long, strap-like muscle, 30–32 cm in length, 5–6 cm in width and 2–3 cm thick. The average muscle fibre length is 24 cm and the tendon of insertion measures an additional 10 cm. The muscle has a dominant vascular pedicle as well as a secondary pedicle or pedicles (Class II), making it suitable for transplantation.[7] The major pedicle is usually the terminal branch of the medial femoral circumflex artery and enters the muscle belly in two or three branches about 6–12 cm inferior to the pubic tubercle. The minor pedicles are usually branches of the femoral artery or the profunda femoris artery and enter the muscle 10–15 cm more distally.[7] The length of the vascular pedicle from its origin to its end measures 6–7 cm and has an external diameter of 1.2–1.8 mm.[7] The venae comitantes of the artery have an external diameter of 1.5–2 mm and a length of 6 cm.[7] Macchi *et al*. in their computed tomography angiography study of 40 patients in 2008, have found that the principle pedicle enters the gracilis muscle at a mean distance of 10 ± 1 cm from the ischiopubic attachment of the muscle and has a mean diameter of 2.5 ± 0.5 mm. The mean number of distal accessory pedicles was found to be 1.8 (range, 1–4) and the artery of the first of these pedicles showed a mean calibre of 2.0 mm.

The gracilis muscle can be split longitudinally into two functional bundles based on the fascicular distribution of the innervations. The advantages of the gracilis muscle as a donor muscle are that it is expendable without any discernable loss of adductor function, the donor defect on the medial thigh is acceptable, the muscle has a Class II vascular pattern making it suitable for transplantation, it has sufficient cross-sectional area making it suitable for functional transfer and it can be harvested endoscopically leaving minimal donor scar and hastening post operative recovery. Although splitting the gracilis muscle longitudinally along its fascicular innervations pattern has been described, use of the transversely split gracilis flap has few references in the literature.[8] While performing a review of the relevant literature, we found only one case report from Austria where the gracilis muscle was split transversely into two halves, each with its own vascular pedicle, and used as two free flaps.[8] Splitting the muscle transversely can be advantageous where there are two small, discontinuous defects that may otherwise require two free flaps for coverage. This technique gives us two small, reliable, well-vascularised free flaps that can be used to cover important structures, fill cavities or increase local vascularity in cases of osteomyelitis. The transversely split muscle, however, cannot be used for functional transfer. The dominant pedicle of the gracilis muscle is reliable and of good calibre, and is almost always found 6–12 cm inferior to the pubic tubercle. A significant correlation between the calibre of the artery of the main pedicle and the volume of the muscle has been found.[9] The calibre of the dominant pedicle artery is significantly larger when originating directly from the femoral artery (45%) (*P* < 0.01).[9] The dominant pedicle of the muscle can support the whole muscle during transfer, but the same cannot be said for the minor pedicles (Class II). However, when the muscle is split transversely, the minor pedicle can reliably support the segment of muscle that it perfuses directly. Thus, one gracilis muscle is turned into twin split-gracilis free flaps by simply splitting it transversely and using the minor vascular pedicle to carry one portion of the muscle.

We have been using the gracilis muscle regularly for small soft tissue defect reconstruction, coverage of small areas of osteomyelitic bone and functional transfer to the palsied hand, with a success rate of 100%. The benefits of gracilis as a donor muscle and its reliable vascular supply have encouraged us to be more liberal in its use in high-velocity trauma of the lower limb for covering vital structures as well as filling soft tissue defects after surgical excision of dead and traumatised tissue. With growing confidence, we are now increasingly using the split gracilis twin free flaps for smaller areas of tissue defects (in lower limb trauma) with gratifying results. It has ensured quick healing and superior results in lower limb trauma and a decreased “down-time” for the patients along with the added benefits of a single donor area and no functional deficit.

We conclude that the transversely split gracilis muscle free flap or the “split gracilis twin flaps” are as reliable as the conventional gracilis free flap and should be considered as a good option where the patient has a need for multiple small free flaps without the need for functional reconstruction. It has the advantages of a “single donor area,” yielding a “single muscle” with “multiple vessels,” which can yield “multiple free flaps,” without the associated co-morbidities of multiple donor areas.
